# When do patients drive after minimally invasive anterior hip replacements? A single surgeon experience of 212 hip arthroplasties

**DOI:** 10.1051/sicotj/2018045

**Published:** 2018-11-22

**Authors:** Ashish Batra, Sophia Gogos, Ikram Nizam

**Affiliations:** Ozorthopaedics, Melbourne, VIC Australia

**Keywords:** Driving resumption, Direct anterior approach, Total hip arthroplasty, Enhanced recovery, Minimally invasive.

## Abstract

*Introduction:* Patients desire to return to normal activities soon after hip arthroplasty, with driving often being an integral component. We aimed to determine when patients resumed driving following a minimally invasive anterior bikini hip replacement and when they returned to work.

*Methodology:* All consecutive patients undergoing elective primary bikini hip replacements between January 2017 and April 2018 were included in the study. Patients who did not drive were excluded. A detailed questionnaire was sent to patients 3–6 weeks after surgery to record their driving status. Fifty patients were randomly selected to assess flexion at the hip, knee and ankle joints while seated in the driver's seat of their own vehicle.

*Results:* Altogether 212 anterior bikini total hip replacements (*L* = 102, *R* = 108 and 1 bilateral one stage) were performed in 198 patients (*F* = 129 and *M* = 69) with a mean age of 69 years. A total of 76% patients returned to driving within the first 3 weeks after surgery, of which 25 (14%) resumed driving within the first post-operative week, 71 (39%) in the second week and 42 (23%) in the third week. Among them, 98.4% stated they were confident when they first started driving and 90.66% stated they were more comfortable driving after surgery than before. Employed patients returned to work within 1–79 days (mean = 24 days).

*Conclusion:* Surgeons may allow patients to resume driving within 1 week after anterior hip replacement and return to work within 3 weeks if they are medically fit and deemed safe.

## Introduction

The primary goals of total hip arthroplasties (THAs) are to relieve pain, improve quality of life and restore mobility [1], determined by the longevity of prosthesis [2] and early return to pre-morbid activities. Prompt resumption of driving [3] is an important surrogate marker of success. To date, there is a lack of medical and legal guidelines regarding the timeline for safe resumption of driving following THA [4]. Current literature reports a minimum 6–8-week period before patients can safely resume driving; however, this is based on outdated studies using posterior THA approaches, where 6–8-week waits are recommended for soft tissue recovery [5,6]. More recently, studies of anterior hip replacements have reported early return to activities [5,7–9], with one study recording brake reaction times reporting a return to preoperative values by day 2 following microinvasive THA. With the advent of the latter technique, patients may be able to resume driving earlier than the previously recommended 6–8 weeks post-operation [10]. With the direct anterior interneuromuscular approach, patients should be able to resume normal activities, including driving, sooner than previously reported and with greater comfort than preoperatively.

The primary aim of this study was to determine when patients first resumed driving without pain following our soft-tissue sparing bikini hip arthroplasty (BHA) [11]. The secondary aim was to determine how soon patients returned to work after anterior THA.

## Methodology

All consecutive patients who underwent elective soft-tissue sparing primary bikini anterior hip replacements [11] by a single surgeon in one institution between January 2017 and April 2018 were included. Informed consent was obtained from patients and the study was approved by the local institutional review board. Non-driving patients, those who had their arthroplasty performed using a different approach, who underwent THAs for acute neck of femur fractures and revision THAs were excluded from the study (*n* = 21). All patients were treated with the same operative technique, perioperative care and post-operative rehabilitation protocol with early mobilisation and discharge to maintain uniformity [7,11].

Patient demographics including age, sex, BMI, hip pathology and operative side were recorded prospectively (Table 1). A detailed questionnaire (Appendix 1) was sent to all patients who underwent a BHA between January 2017 and April 2018, 3–6 weeks after their procedure. Patients were reviewed 2 weeks post-operation and again at 6–8 weeks post-operation. Their driving status at both reviews was recorded in the patient notes.

**Table 1:** Patient demographics.

Characteristic	*N* = 198	%
*Age (years)*		
Mean	69	
Range	46–91	
*BMI*		
Mean	28.10	
Range	17.63–56.44	
*Gender*		
Female	129	(65.2)
Male	69	(34.8)
*Operation*		
Right THA	108	(51.23)
Left THA	102	(48.3)
Bilateral THA	1	(0.47)
*Vehicle type*		
Automatic	162	(81.8)
Manual	12	(6.1)
Unknown	24	(12.1)

At the 2-week post-operative review, 50 patients from the study were randomly selected to assess flexion at the hip, knee and ankle joints while seated in the driver's seat of their own vehicle. Randomisation was performed via patient surnames de-identified and entered into a random number generator. A smaller patient sample was used to assess joint flexion due to the increased complexity of undertaking assessments across multiple institutions and limitations of necessary equipment and personnel.

Two measurements of flexion of each joint were recorded with the patient seated in their personal vehicle. Measurements were taken with a goniometer by one research assistant at a single appointment. An average of the two measurements at each joint was calculated to minimise random error and ensure values were representative of respective joints.

### Surgical procedure

All operations were performed using BHA technique previously described [11], which included both cemented (CPCS Smith and Nephew, Memphis TN) and un-cemented femoral components (Polar Smith and Nephew AG, Baar, Switzerland). Femoral head (Oxinium Smith and Nephew, Memphis TN) sizes used included 28 mm (1.5%), 32 mm (72.5%) and 36 mm (26%). The femoral head size was determined by cup size. Acetabular shells of 48 mm (Acetabular shell: R3 three-hole HA-coated Smith and Nephew Memphis, TN) were used for femoral heads less than or equal to 28 mm in diameter. Larger femoral heads (>36 mm) were encased in 52 mm shells. Skin closure was achieved using Monocryl monofilament absorbable sutures and a thin Comfeel dressing applied.

### Statistical analysis

Ranges and means were calculated for all outcome measures using responses to the driving questionnaire distributed to patients. Correlation between resumption of driving and multiple outcome measures was undertaken using a chi-squared test.

## Results

**Figure 1:**
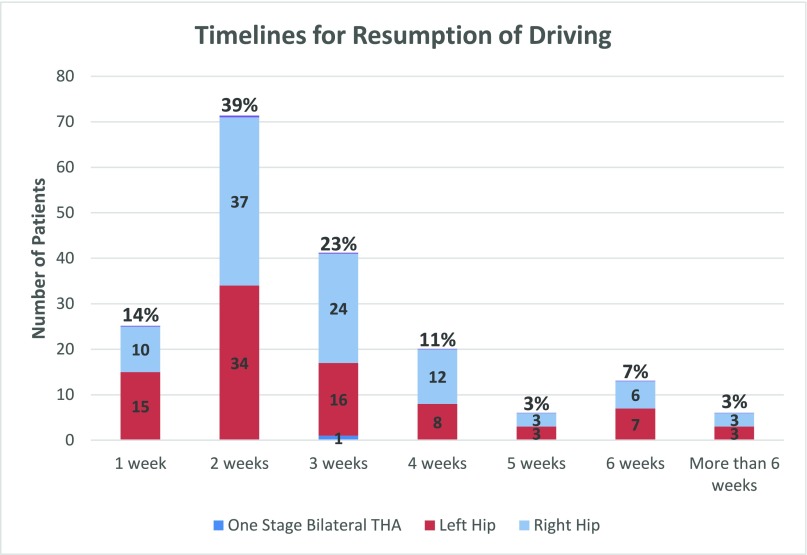
Patient timelines for the resumption of driving following BHA.

In total, 138 (76%) patients returned to driving within the first 3 weeks after surgery, of which 25 (14%) patients resumed driving within the first post-operative week, 71 (39%) patients drove in the second week and 42 (23%) returned to driving in the third week (Figure 1). The remaining 45 patients reported that they could have driven earlier but chose not to as they had preferred alternatives. The earliest resumption of driving was on the 2nd day post-surgery (*n* = 2). There were 179 (98.35%) patients who stated that they were confident when they first resumed driving. There were 29 patients (13.7%) who did not return to driving within 8 weeks post-surgery, 1 patient due to medical comorbidities and the remaining 28 relied on family for transport; however, they were confident that they could have driven themselves if needed.

Although more patients with left-sided THA resumed driving in the first post-operative week than patients who underwent right-sided THA (*n* = 14, *n* = 9, respectively), there was no significant difference in the time taken to resume driving between operative side (*x*^2^ = 3.37, *p* = 0.50). About 90.66% of patients stated that they were more comfortable driving post-surgery than before surgery as their arthritic hip pain and stiffness was eliminated, thus enabling easy entry and exit out of the vehicle. Over 92% were climbing stairs independently before driving, while the remaining patients used side rails. Nearly 82% of patients drove automatic cars and 6% were manual drivers, with the remaining 12% not completing the applicable question. There was however no significant correlation between time taken to resume driving and transmission of vehicle driven by the patient (*x*^2^ = 0.013, *p* = 0.91). About 29.8% of patients were still working at the time of their operation, and the remainder were retired. Of the working patients, the average number of days taken to return to their usual work at any capacity was 24 days (range = 1–79 days).

About 49.5% of our patients were mobilizing well before driving without any walking aids (Table 2). Of the remaining 50.5% (92 patients), 74% (68 patients) were using one crutch only. All patients tested the car brake before resuming driving, with 3.85% of all patients reporting they did not feel confident with emergency braking in the 6 weeks following their operation. Besides, 16.5% of patients reported they felt pain while driving, although they also stated that the pain was mild and did not distract them. Another 1% stated they felt somewhat drowsy during their initial drive following surgery and thus delayed driving for a further week. Patients were given clear instructions to avoid narcotics upon resuming driving before discharge from hospital. There were no dislocations, infections or thromboembolic events in this patient group. One patient had a revision THA following a significant fall down stairs 23 days following their initial operation. There were no motor vehicle accidents (MVA) or near misses reported by patients during follow-up consultations or reported in the driving questionnaire.

**Table 2:** Patient responses to driving questionnaire.

Questions asked in questionnaire	Yes	No
Walking aids used when resumed driving	50.55%	49.45%
Ability to climb stairs when resumed driving	92.31%	7.69%
Confident to perform emergency braking if needed	96.15%	3.85%
Confident driving the first time after surgery	98.35%	1.65%
Comfort driving post-surgery as compared to pre-surgery	91.21%	8.79%
Ability to get in and out of the car comfortably	90.66%	9.34%
Pain while driving	16.48%	83.52%
Pain or discomfort as a distraction from driving	2.75%	97.25%
Feelings of drowsiness or ill-prepared to continue driving	1.10%	98.90%

**Figure 2:**
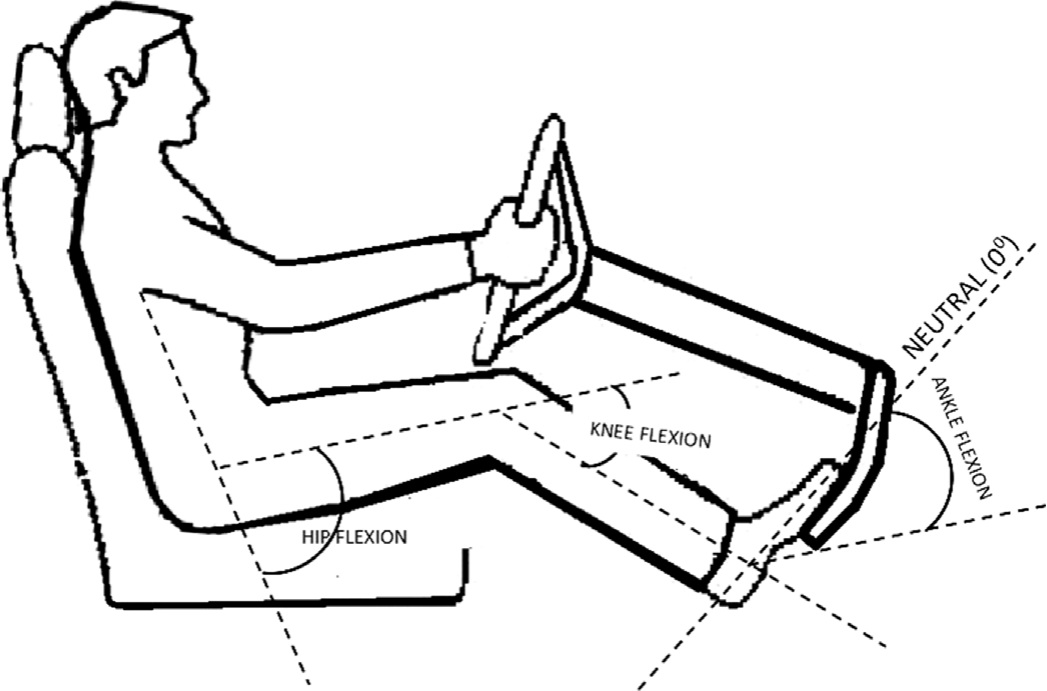
Demonstration of flexion angle measurements of hip, knee and ankle joints.

**Figure 3:**
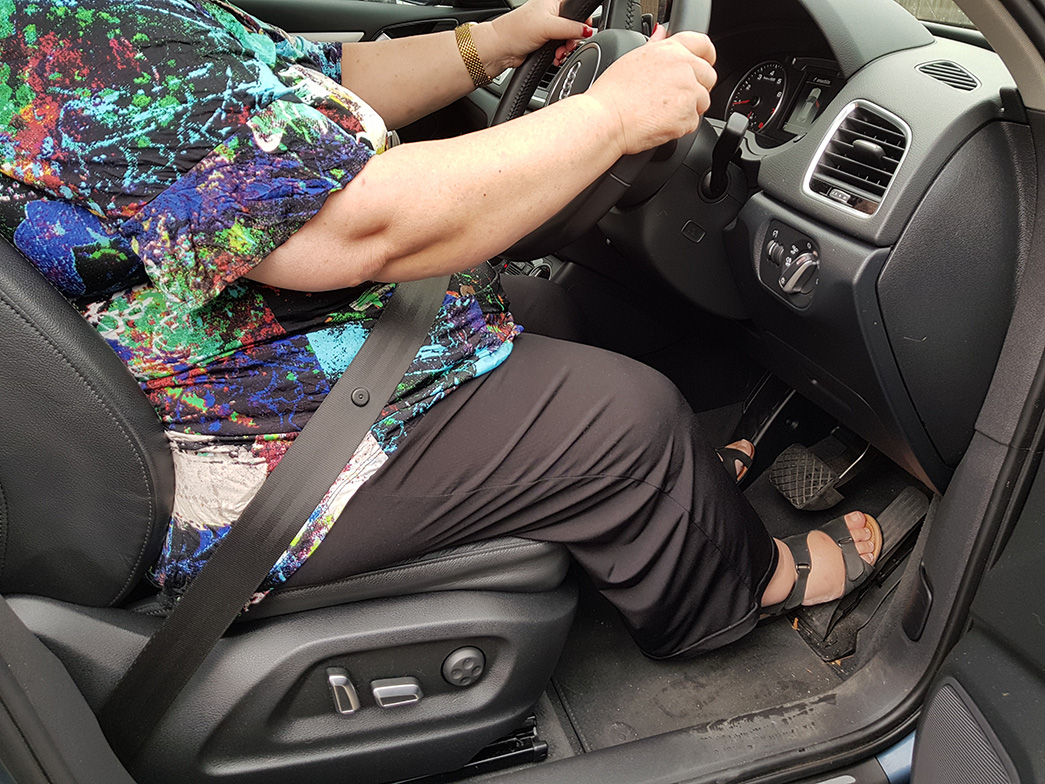
Patient in driving seat day after anterior THA, with hip flexion (70°), knee flexion (45°) and ankle plantar flexion (30°) while accelerating.

**Figure 4:**
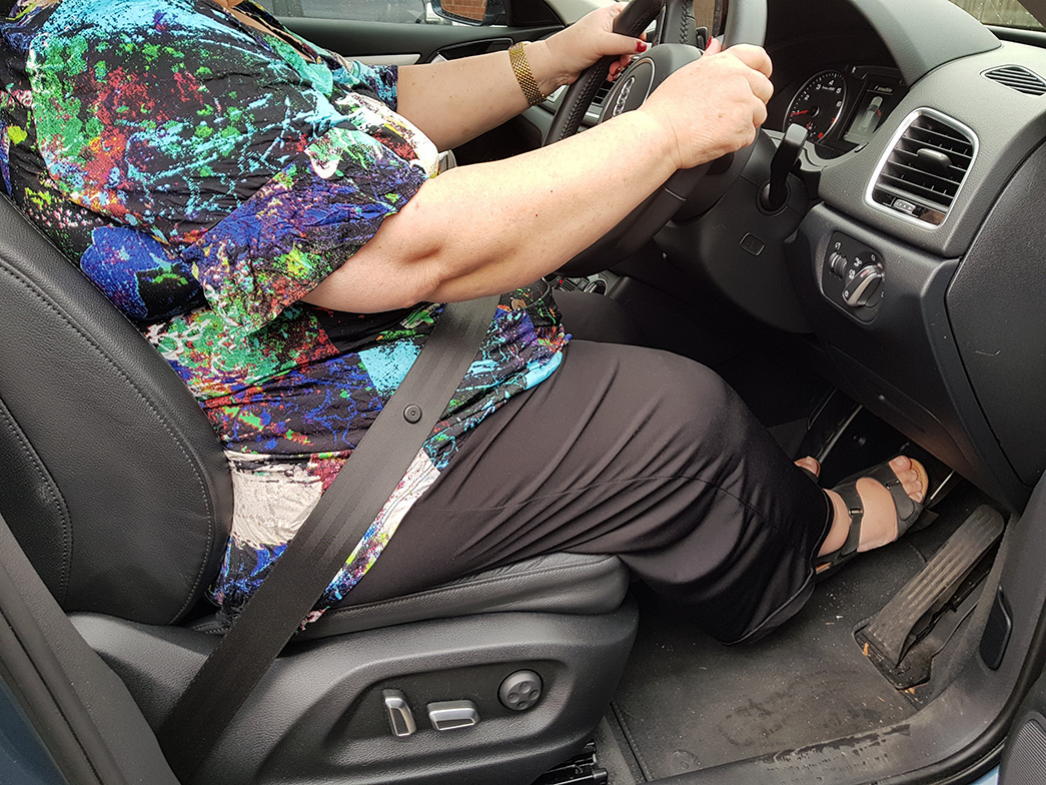
Patient in driving seat 2 weeks after anterior THA with hip flexion (72°), knee flexion (40°) and ankle in neutral position while braking.

The measurements of angles of flexion at hip, knee and ankle during simulated acceleration and emergency braking (Figure 2) among 50 patients demonstrated that ankle movements appear to affect driving more than hip and knee movements. While accelerating, patients required a range of 0–43° ankle plantar flexion; while braking, majority of patients had their ankle in 0–10° plantar flexion (Figures 3 and 4). Majority of movements while accelerating or braking are at the ankle joint, although the knee joint is predominantly engaged while braking, with 0–5° of knee extension. During emergency braking, the ankle plantar flexion range may rise to 30° as the driver uses maximal force to compress the brake completely. Hip and knee movements required an average of 71° (66°–76°) and 53° (38°–70°) flexion, respectively, in the simulated positions. Hip adduction and internal rotation of up to 5–10° was noted when patients shifted their right lower extremity from the accelerator to the brake in automatic vehicles.

## Discussion

Our study demonstrates that patients were able to resume driving several days after undergoing soft tissue sparing BHAs [11]. Currently, there are minimal studies evaluating the resumption of driving following a total hip replacement. Our study is the largest single surgeon analysis to date evaluating the actual timeline of resumption of driving following anterior total hip replacements.

The American Academy of Orthopaedic Surgeons recommends a waiting period of 4–8 weeks post-surgery before recommencing driving in an automatic car. Current advice for drivers of manual vehicles and patients of right-sided THA is not as clearly defined in the literature. In either case, patients should seek the advice of a medical professional including their treating surgeon before resuming driving.

A study by Qurashi et al. [10] evaluating driving after total hip arthroplasty in 100 patients concluded that break response times (BRT) reached preoperative values by day 2 following surgery. Consequently, patients may be able to recommence driving sooner than the previously recommended 6 week post-operation. Ganz [12] demonstrated a return of BRT to preoperative values for right-sided THA at 4–6 weeks post-surgery, and MacDonald and Owen [3] assessed their patients 8 weeks after surgery. For left-sided THA, both Ganz [12] and MacDonald and Owen [3] demonstrated a statistically insignificant alteration of BRT when driving was resumed in a vehicle with automatic transmission. These studies suggested that driving may be resumed as soon as one week after surgery, depending on post-operative pain.

Our patients recommenced driving from week one after surgery. It is possible that the soft-tissue sparing operative approach with enhanced recovery program contributed to the early return to driving. Over 91% of patients reported it was much more comfortable to drive after surgery than before, as the arthritic pain and stiffness was eliminated almost immediately. Our study relies on the patient's experience in their own vehicle, rather than utilising an automatic car simulator to evaluate BRT, which does not accurately mimic natural driving conditions. Additionally, the questionnaire which was utilised comprises multiple aspects of driving activity, including entering and exiting the vehicle, braking and pain as a distraction. More importantly, measuring hip, knee and ankle plantar flexion angles required for acceleration and braking demonstrated that ankle movements seem to affect driving more than hip or knee movements. While accelerating, patients required a range of 0–43° ankle plantar-flexion, and while braking majority of patients have their ankle in 0–10° plantar flexion. Similarly, hip and knee movements required an average of 71° (66°–76°) and 53° (38°–70°) flexion, respectively, which facilitates the resumption of driving post-THA, if pain is minimal.

There are no validated questionnaires pertaining to driving after joint replacements in the literature. We designed our questionnaire (Annexure 1) considering safety as a priority and included practical aspects patients would consider before driving. The senior surgeon allowed a patient to drive only after post-operative assessment if the patient was medically fit and confident, walking pain free with or without a single walking aid, able to get in and out of a car comfortably, not taking oral narcotic analgesia and if accompanied by a passenger on the first driving occasion.

A study by Abbas and Waheed [4] reported 105 of 130 patients who underwent THA were able to resume driving between week 6 and 8. Of the remaining 25 patients, 22 returned to driving at 12 weeks and 3 were not confident driving at 12 weeks post-THA. It was concluded that the time taken to resume driving was dependent on patient's recovery and confidence in their own ability. Due to the subjective nature of this report, a time frame could not be applied to the general population of THA patients. Contrary to this, we found that 25 (14%) of our patients started driving in the first week post-surgery, 71 (39%) in the second week and 42 (23%) in the third week. Two patients drove after the third week, but only because they were in rehabilitation post-surgery, both reported they could have driven earlier. Furthermore, the majority of our patients felt confident and less apprehensive when recommencing driving, potentially due to enhanced recovery measures which were taken including the minimally invasive surgical technique, local analgesia infiltration and early mobilisation post-procedure. 1.65% patients stated that they were not confident to drive the initial time post-surgery, which caused them to delay their resumption to driving by 1–2 weeks, although all eventually drove with confidence.

Previous studies evaluating the burden of large joint replacement surgery on returning to work demonstrate that there is a significant psychosocial impact of prolonged absence from work following hip arthroplasty [13]. After BHA, patients were able to mobilise early and resume driving to work, thus minimising these potential consequences of prolonged absence. Although 139 patients were retired, resuming driving was important to maintain their independence and resume outdoor activities and routines, as noted by our patients in the questionnaire.

All patients were seen by a surgeon and physician post-surgery before discharge. None of our patients have directly or indirectly been involved in any motor vehicle accidents within the 6-week post-operative period, nor did they report any adverse events.

There is a potential recall bias that must be considered due to the time period between patients receiving the questionnaire and the date of their operation. However, results reported in the questionnaires were cross-referenced with clinical notes recorded at the standard 2- and 8-week post-operative appointments. Additionally, there was no uniformity in the type of vehicle assessed in the study, but this may more accurately mimic the variety of automobiles including automatic, manual, SUV, sedans, trucks, driven by the general population. As ideal seating positions are dependent on patient factors such as level of comfort, there were a wide range of values reported for flexion at hip and knee. Finally, our questionnaire has not been validated, although it was designed using practical and relevant questions that are easily reproducible for future research.

## Conclusion

Our findings demonstrate it is feasible and safe to resume driving within one week following a soft-tissue sparing anterior bikini hip replacement, irrespective of the side of surgery. Working patients returned to employment within 3 weeks post-operative, provided they were medically fit. Patients with complex medical comorbidities and those taking narcotics should seek the advice of their treating surgeon before resuming driving or returning to work.
